# Flexible Piezoresistive Pressure Sensor Based on Electrospun Rough Polyurethane Nanofibers Film for Human Motion Monitoring

**DOI:** 10.3390/nano12040723

**Published:** 2022-02-21

**Authors:** Bin Xue, Haiyi Xie, Jinxu Zhao, Jianming Zheng, Chunye Xu

**Affiliations:** Hefei National Laboratory for Physical Sciences at the Microscale, Department of Polymer Science and Engineering, University of Science and Technology of China, Hefei 230026, China; binxue@mail.ustc.edu.cn (B.X.); xiehy@mail.ustc.edu.cn (H.X.); zhaojinxu@mail.ustc.edu.cn (J.Z.)

**Keywords:** piezoresistive pressure sensor, electrospinning, microstructure, PU/Ag film, nanofibers

## Abstract

Flexible piezoresistive pressure sensors have been attracted a lot of attention due to their simple mechanism, easy fabrication, and convenient signal acquisition and analysis. Herein, a new flexible piezoresistive sensor based on microstructured electrospun rough polyurethane (PU) nanofibers film is assembled. The microstructured PU film with tiny bumps is prepared in one step via electrospinning technology, which imparts a microstructured rough upper surface and a smooth lower surface. With this unique microstructure, we have made it possible for PU/Ag films to serve as sensing layers for piezoresistive sensors by introducing a silver conductive layer on the surface of electrospun PU film. The fabricated piezoresistive pressure sensor delivers high sensitivity (10.53 kPa^−1^ in the range of 0–5 kPa and 0.97 kPa^−1^ in the range of 6–15 kPa), fast response time (60 ms), fast recovery time (30 ms), and long-time stability (over 10,000 cycles). This study presents a fabrication strategy to prepare the microstructured PU nanofiber film using electrospinning technology directly, and the as-developed sensor shows promise in applications such as wrist pulse measurement, finger movement monitoring, etc., proving its great potential for monitoring human activities.

## 1. Introduction

Recently, researchers have paid more attention to flexible sensors because of the development of artificial intelligence, the internet of things, human-machine interfaces, etc. Flexible sensors can be widely used in many fields such as electronic skin, human health monitoring, soft robotics, and wearable and portable devices [1,2,3,4,5,6]. To match various applications, flexible sensors of various types (strain, temperature, humidity, gas, pressure sensors, etc.) have been developed [7,8,9,10]. Among them, pressure sensors have attracted a lot of interest due to the significance of pressure in daily life and industry application. Compared to the rigid pressure sensors, flexible pressure sensors based on flexible substrates and sensing layers are more suitable in practical applications.

Pressure can be converted into readable electrical signals and output by pressure sensors. To date, a lot of pressure sensors based on different sensing mechanisms have been designed. The most common sensing mechanisms reported previously are piezoelectric sensing [11,12], triboelectric sensing [13,14], capacitive sensing [15,16], piezoresistive sensing [17,18,19], and dual-mode sensing [20]. Among them, piezoresistive pressure sensors have gained increasing attention from many researchers due to their simple mechanism, facile fabrication, and convenient signal collection and analysis. Generally, the response of a piezoresistive-type sensor to applied pressure is presented as a change in the resistance of its conductive layers, caused by a variance of contact area between different conductive layers. Hence, the conducting area or number of conductive pathways usually plays a key role in the pressure-sensing performance of piezoresistive-type pressure sensors. A typical strategy to enhance the pressure-sensing performance was introducing 3D microstructures on the films comprising the sensors [21,22]. The existence of microstructures could lead to an increased change in contact area or larger structural deformation under pressure.

To produce the microstructures on the surface of flexible films, various surface engineering methods have been investigated. A common approach is to design surface structures (e.g., micropyramids, microdomes, microgrooves, etc.) by taking advantage of an expensive photolithography technique. The common method is to fabricate micromolds by photolithography first and then transfer the conductive elastomer to the micromolds. Finally, the cured elastomer film is removed from the mold to obtain a highly customizable microstructured film. For example, researchers reported a flexible conductive composite film with microdome arrays by micromolding a mixture of carbon nanotubes (CNTs), base agent, and curing agent of polydimethylsiloxane (PDMS) with a silicon mold. They prepared the piezoresistive sensor by combining the above two composite films with patterned sides facing each other and the sensor exhibited potential applications in human-health monitoring [23]. Another approach is the utilization of existing materials, such as sandpapers [24], leaves [25], silk [26], etc. These materials can be used as molds due to their unique surface microstructures. Jian et al. reported a pressure sensor based on aligned carbon nanotubes/graphene (ACNT/G) films and microstructured PDMS films molded from an Epipremnum aureum leaf [27]. This high-performance sensor demonstrated high sensitivity, a low detection limit, high stability, and versatile applications in the detection of different types of forces. However, sometimes these methods are complex, multi-step, expensive, and time-consuming. It is still necessary to explore more methods to introduce microstructures into the films comprising the sensors. To the best of our knowledge, a microstructured electrospun PU nanofiber film prepared directly via electrospinning technology for a piezoresistive pressure sensor has been rarely reported.

In this research, a microstructured PU nanofiber film with tiny bumps is prepared by a one-step electrospinning process and used to assemble a flexible pressure sensor. The electrospun PU film has a rough upper surface and smooth lower surface. The presence of this unique morphology potentially allows the film to serve as a sensing layer in a piezoresistive pressure sensor. The PU/Ag films prepared by uniformly coating the silver layer on the upper surface of the electrospun PU film have abundant sensitive sites and high flexibility, contributing to the high performance of the pressure sensor. The fabricated pressure sensor is assembled by facing two PU/Ag films to each other with PDMS substrates and exhibits high sensitivity and good stability. This study presents a new strategy to introduce tiny bumps on electrospun PU films which serve as pressure-sensitive layers in the pressure sensor. The fabricated sensor shows promising applications for human motion monitoring.

## 2. Materials and Methods

### 2.1. Materials

Polyurethane (PU) was purchased from Wanhua Company (Shanghai, China). N, N-dimethylformamide (DMF) was bought from Sinopharm Chemical Reagent Co., Ltd. (Shanghai, China). The base and the curing agent of PDMS were obtained from Dow Chemical Company (Midland, MI, USA) (Sylgard 184). The mixed ratio of base and curing agent was 10:1. All of the chemicals were used without further purification.

### 2.2. Preparation of the Electrospun PU Film and PU/Ag Film

PU solution was prepared by dissolving 5 g polyurethane into 20 mL DMF with magnetic stirring and heating at 80 °C for 6 h. After cooling to room temperature, the homogeneous PU solution was transferred into a 20 mL syringe with a stainless flat-end steel needle. The whole electrospinning process was completed in a nanofiber electrospinning unit (Kato Tech Co., Ltd., Kyoto, Japan) with a high voltage power supply, a syringe pump, and a grounded cylindrical collector. The electrospinning unit is displayed in Figure S1. The cylindrical collector covered with aluminum foil was placed 8 cm away from the steel needle tip. A high direct current voltage of 8.5 kV was applied between the needle tip and the cylindrical collector, and the flow rate of the PU solution was 1 mL/h. During electrospinning, the cylindrical collector remained stationary, and the rotation speed was 0. The traverse speed was about 4 cm min^−1^. A rough PU nanofiber film was obtained on the collector after electrospinning for 90 min. Finally, the conductive silver paste was coated uniformly on the surface of the PU nanofibers film to serve as the conductive layer. Briefly, a few drops of silver paste were dropped on the upper surface of the PU nanofibers film, and then the silver paste was painted back and forth several times on PU nanofibers film using a brush. The average amount of silver paste was 0.01 g cm^−2^. The PU/Ag film was dried at 80 °C for 30 min to finish the curing of the silver paste.

### 2.3. Preparation of PDMS Substrate

For the assembly of the piezoresistive pressure sensor, two PDMS sheets (2 cm × 2 cm) were prepared using a polytetrafluoroethylene (PTFE) mold and used as the substrates. Briefly, the base and the curing agent of PDMS were mixed and stirred well, and the mixed PDMS solution was vacuumed to degas for 30 min. PDMS thin film was obtained by casting 0.3 g mixed solution into the commercial PTFE mold, and the mixed solution was cured at 80 °C for 1 h. Finally, the PDMS film with a thickness of about 0.6 mm was peeled off from the mold for future use.

### 2.4. Assembly of the Piezoresistive Pressure Sensor

The PU/Ag film was cut to the desired size (1.5 cm × 1.8 cm). Conductive aluminum tapes were fixed on the PDMS substrate, then two PU/Ag layers were transferred to the two PDMS substrates and placed facing each other. The conductive tapes were connected to the silver layer on the PU/Ag film by silver paste. The PDMS substrates encapsulated the PU/Ag layers into an integrated sensor. The PDMS substrate, PU/Ag film and piezoresistive pressure sensor exhibit good flexibility and can be bent without fracture (Figure S2).

### 2.5. Characterization

A scanning electron microscope (SEM) (SU8220, Hitachi, Tokyo, Japan) was used to observe the surface morphologies and cross-sections of the fabricated films. Energy-dispersive X-ray spectrometry (EDX) in conjunction with SEM was used to investigate the distribution of elements of the PU/Ag film. The cross-sections of the samples were prepared by quenching the samples using liquid nitrogen. All the samples for the SEM test were fixed on the sample holder with carbon tape. The loading of pressure was performed with a universal testing machine (SUNS UHM-2102) to evaluate the performance of the piezoresistive pressure sensor. A digital multi-meter (34410A, Agilent Technologies, Santa Clara, CA, USA) was used to record signals of resistance in the application section, and the current–voltage curves of the fabricated sensors were measured through an electrochemical workstation (CHI 660D, Chenhua, Shanghai, China).

## 3. Results and Discussions

### 3.1. Fabrication Process of the Piezoresistive Pressure Sensor

**Figure 1:**
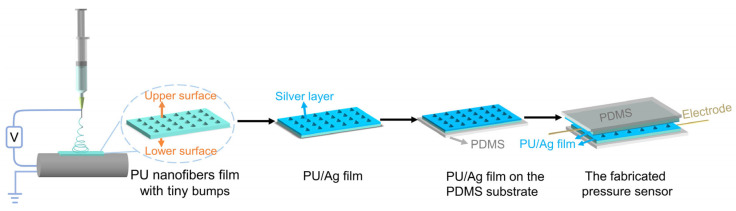
A schematic diagram of the fabrication process of the piezoresistive pressure sensor.

The basic steps of the fabrication of the piezoresistive pressure sensor in this research are schematized in Figure 1. Firstly, the rough electrospun PU nanofibers films with tiny bumps were prepared through the electrospinning method. Different from the methods proposed previously to produce microstructures into pressure sensors such as pre-designed molds and using naturally existing materials as molds, the tiny bumps on the PU nanofibers film appeared directly during the electrospinning process, resulting in the surface microstructure of the electrospun PU film. The electrospun PU film was removed from the aluminum foil and cut into the desired size after the whole electrospinning process. Then, the silver layer was introduced onto the rough side of the film to serve as a conductive layer. After the preparation of the PU/Ag film, two PU/Ag films as sensing layers were transferred to the PDMS substrate and eventually assembled into a piezoresistive pressure sensor. The final assembled pressure sensor is capable of sensing loading pressure through the detection and output of the change in resistance between the two sensing layers.

### 3.2. Morphologies of the Electrospun PU and PU/Ag Film

**Figure 2:**
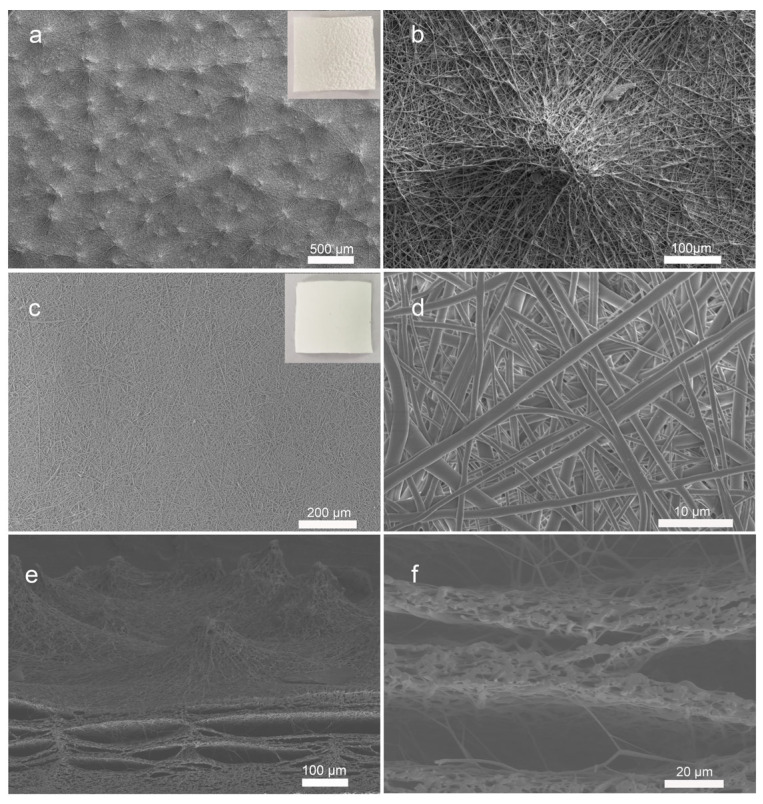
SEM images of the electrospun PU nanofibers film: upper surface at low magnification (**a**) and high magnification (**b**), lower surface at low magnification (**c**) and high magnification (**d**), and a cross-section at low magnification (**e**) and high magnification (**f**). The insets are photos of the upper surface and lower surface of the electrospun PU film, respectively.

The PU nanofiber film was fabricated directly using a facile and repeatable electrospinning technology and the morphology of the electrospun PU film was examined by scanning electron microscopy as shown in Figure 2. In contrast to conventional electrospun nanofiber films, the as-prepared PU nanofibers film presents a completely different surface morphology on its upper surface (Figure 2a,b) and lower surface (Figure 2c,d). The upper surface is the side that is away from the collector, while the latter is attached to the aluminum foil. The former exhibits a rough surface morphology with tiny bumps formed by nanofibers, and the latter presents a smooth surface. During the electrospinning process, PU nanofibers were randomly overlaid. A lot of tiny bumps could be observed on the upper surface of the film. The existence of tiny bumps results in a rough surface microstructure of the PU film. From the SEM image of a single tiny bump, as shown in Figure 2b, it is clear that the mountain-type tiny bump with a diameter of about 200 μm is composed of twisted PU nanofibers. Simultaneously, the electrospun PU film presents a smooth lower surface. The diameters of PU nanofibers are about 1 μm. An SEM image containing both the upper surface and lower surface is presented in Figure S3.

The introduction of microstructure into the piezoresistive pressure sensors is a conventional strategy to improve their pressure-sensing performance. Compared with the existed pressure sensors with complicated fabrication technology used in other studies, the microstructure PU nanofibers film can be manufactured in one step by electrospinning. To the best of our knowledge, no study in the literature reports a similar phenomenon. The existence of the tiny bumps may be derived from the influence of residual solvent in the electrospinning process. Usually, most of the solvent in the polymer solution evaporates when the jet moves toward the collector in the electrospinning process, but some solvent remains on the collector for further evaporation. In this research, the nanofibers were deposited in a small area as the cylindrical collector remains stationary, as shown in Figure S4. When the rate of solvent accumulation exceeds the rate of its evaporation, as the residual solvent increases, the charges accumulate in some regions of the surface of the electrospinning film. These regions may change the direction of the jet, which leads to uneven deposition of PU nanofibers and generation of the tiny bumps on the upper surface of the electrospun PU film. Meanwhile, all of the PU exhibits fibrous morphology, which can be confirmed from the enlarged cross-section SEM image in Figure 2e,f.

**Figure 3:**
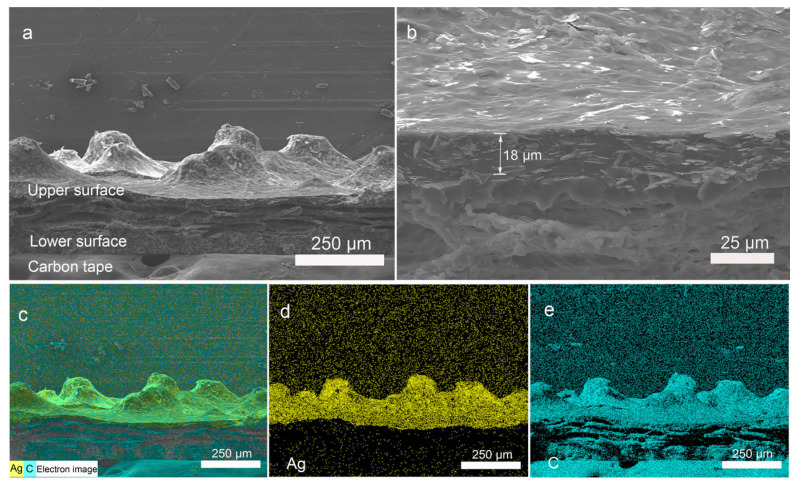
Cross-section SEM images of the PU/Ag film: (**a**) low magnification; (**b**) high magnification. Energy-dispersive X-ray spectroscopy (EDX) mapping of PU/Ag film, including EDX layered image (**c**), element Ag (**d**), and element C (**e**).

A silver layer was selected as the conductive layer on the electrospun PU film owing to its high conductivity. To investigate the distribution of the silver layer, the cross-sectional morphology of the PU/Ag film was observed with SEM. The cross-section SEM images of the film with different magnifications are shown in Figure 3a,b. It can be seen that the silver layer is uniformly distributed on the surface of the electrospun PU film and covers these tiny bumps. The thickness of the silver layer is about 18 μm. With a higher magnification cross-section SEM image, the boundary between the silver layer and the PU nanofibers layer is discernible, which is consistent with the distribution of silver element in the EDX layered image shown in Figure 3c. A cross-section SEM image of PU/Ag film which shows the PU nanofibers without silver contact is also presented in Figure S5. To further illustrate the distribution of the silver layer, the EDX mapping analysis was also performed. The relevant results are presented in Figure 3c–e. The elements Ag and C were chosen as representative elements to show their elemental distribution in the PU/Ag film. An EDX layered image of elements Ag and C is also shown. The elemental mapping analysis of the prepared film demonstrates the effective introduction of the silver layer during the fabrication process and its uniform distribution on the rough surface of the PU/Ag film.

### 3.3. Sensitivity and Working Mechanism

**Figure 4:**
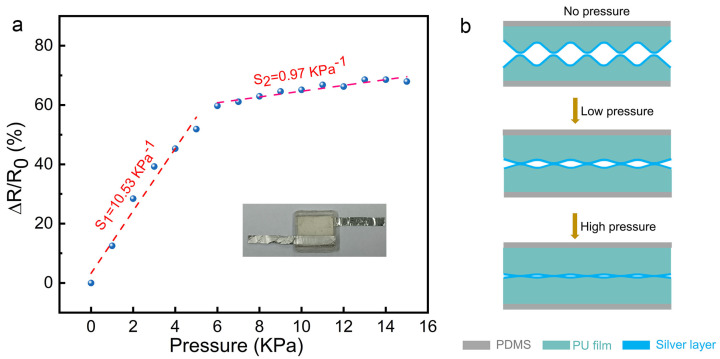
(**a**) Relative resistance change versus applied pressure on the flexible piezoresistive pressure sensor. Inset: a photo of the assembled pressure sensor. (**b**) The working mechanism of the fabricated piezoresistive sensor under loading pressure.

Based on the rough surface of the PU/Ag films, a piezoresistive type pressure sensor was fabricated by assembling two of the microstructured PU/Ag films with the rough sides facing each other, as displayed in Figure 4a. PDMS sheets were chosen as the substrates. To evaluate the sensing performance of the fabricated sensor, we measured the relative resistance change versus loading pressure of the flexible pressure sensor. The sensitivity of the fabricated sensor can be calculated by fitting the experimental data shown in Figure 4a, which is defined as:

(1) $${\Delta R=R}_{0}\;-\;R$$

(2) $$S=\frac{{\delta (\Delta R/R}_{0})}{\delta P}$$

where R_0_ is the initial resistance, R is resistance with the applied pressure, and P is the loading pressure.

As can be seen in Figure 4a, the values of ΔR/R_0_ show an upward trend, indicating a gradual decrease in the resistance of the sensor as the loading pressure increases. The curve can be roughly divided into two regions, corresponding to two different sensitivity values. We obtained a high sensitivity of about 10.53 kPa^−1^ in the low-pressure range (0–5 kPa) and a reduced sensitivity of 0.97 kPa^−1^ in the high-pressure range (6–15 kPa) from the two fitted lines, which is higher than or comparable to the previous reported piezoresistive pressure sensors. A summary of the existing piezoresistive pressure sensors is shown in Table 1. It is worth noting that all sensitivities are calculated based on the relative resistance change (ΔR/R_0_ (%)). In addition, a comparison of other types of sensors reported recently with the proposed sensor is listed in Table S1.

The working mechanism of the as-prepared piezoresistive pressure sensor is the change of the contact resistance between two microstructured films (Rc) in response to loading pressure. When pressure is applied to the piezoresistive sensor, the distance between two fabricated PU films decreases, and the contact area increases, resulting in the formation of more conductive pathways and thus a decrease in Rc, as illustrated in Figure 4b. On unloading, the microstructured PU films recover to their initial state, and the newly formed conductive pathways are broken, therefore, leading to the increase in the resistance. As a result of the aforementioned investigation, two different sensitivity values in different pressure ranges were obtained. High sensitivity in the low-pressure range may be attributed to the introduction of the microstructures on the surface of electrospun PU films. The presence of microstructures provides the sensor with more space for deformation under pressing. The apparently reduced sensitivity in the high-pressure region may be caused by saturation of the deformation of the microstructure.

### 3.4. Pressure Sensing Performance

**Figure 5:**
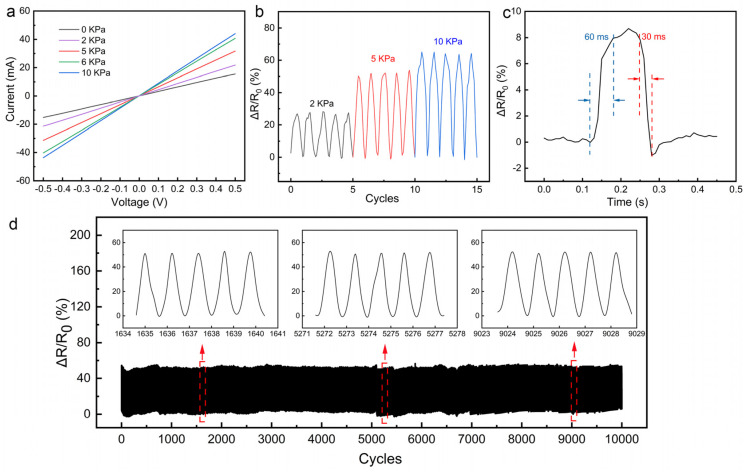
(**a**) I–V curves of the fabricated piezoresistive pressure sensor under different pressures; (**b**) relative resistance changes of the pressure sensor versus cyclic loading of 2 kPa, 5 kPa, and 10 kPa; (**c**) response and recovery time of the pressure sensor under a gentle touch; (**d**) cycling stability of the as-prepared sensor over 10,000 cycles under 5 kPa.

The current–voltage (I–V) curves of the as-prepared sensor under different loading pressure are shown in Figure 5a. The I–V curves exhibit high linearity, which is in accordance with Ohm’s law. The current under the same voltage increases with the increasing loading pressure as a result of decreasing resistance. The cyclic pressure-sensing performance of the fabricated sensor was also investigated, and the result is shown in Figure 5b. The sensor output stable and repeatable signals during the cycling process, demonstrating its reliability in discriminating between various pressures. Figure 5c is a typical signal of the fabricated sensor loading pressure. The response time and the recovery time are about 60 ms and 30 ms, respectively, indicating the rapid responsive characteristic of the sensor to the pressure. The stable response behavior to loading pressure with a short response time (60 ms) enables the monitoring of fast pressure signals, which implies potential applications in human motion monitoring. To further confirm the long-term stability of the fabricated piezoresistive senor, a continuous loading–unloading cycling test was performed. The sensor exhibits a stable response of the loading pressure over 10,000 cycles (Figure 5d), and the peak values of the relative resistance change are almost the same during the cycling test. This may be attributed to the high flexibility and elasticity of both PDMS and pressure-sensitive PU/Ag layers.

### 3.5. Applications of the Fabricated Sensor

**Figure 6:**
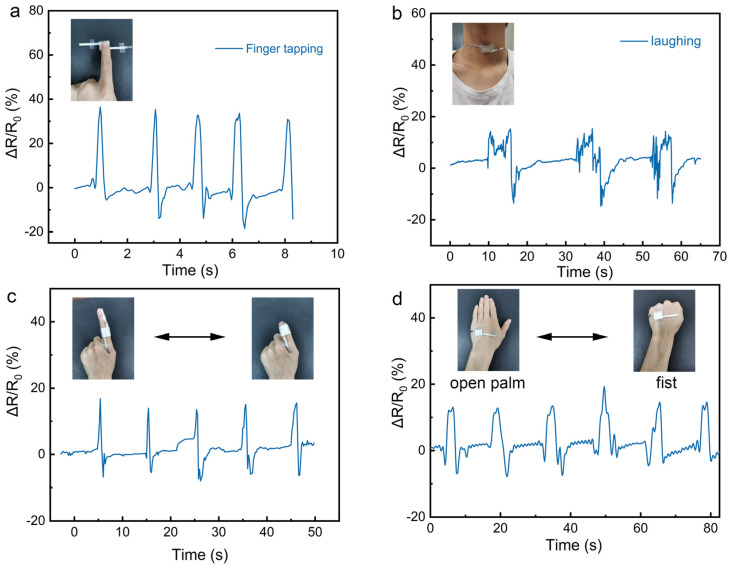
Applications of the electrospun PU film-based pressure sensor to human motion detection. Real-time signal of the pressure sensor in monitoring motions of (**a**) finger tapping, (**b**) laughing, (**c**) finger bending, and (**d**) a posture of a human hand. The insets are the photos of the tester wearing the pressure sensor.

Due to the flexibility, high sensitivity, stability, and fast response speed of the electrospun PU film-based piezoresistive pressure sensor, it could be a promising candidate for real-time human motion monitoring. As shown in Figure 6a, the real-time relative resistance change of finger touch was investigated. The pressure sensor shows a fast response during the finger-tapping process, which allows for the monitoring of the activity of fingers. As a demonstration, the sensor was also attached to a human throat (Figure 6b). It can clearly detect the vibration of the vocal cords when the tester laughed. The results illustrate that our fabricated sensor exhibits great potential for application in monitoring activity near the throat. The sensor was also used to test the bending of a human index finger (Figure 6c). When the index finger starts to bend, the sensor is compressed, resulting in more conductive pathways and a decrease in resistance. The fabricated sensor was also fixed on the back of the tester’s hand to detect the posture of the hand, as shown in Figure 6d. The tester repeatedly opened the palm and clench the fist, and the relative resistance change of the piezoresistive pressure sensor was recorded. The sensor shows a stable and rapid response to the posture of the tester’s hand. The above results indicate the potential capability of the electrospun PU film-based sensor in monitoring human motion.

**Figure 7:**
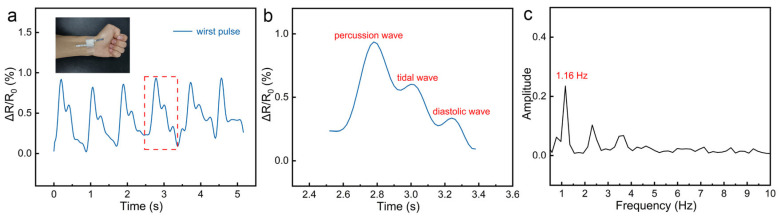
(**a**) Monitoring of the wrist pulse based on the fabricated piezoresistive pressure sensor. The inset is a photo of the wrist of the tester with the pressure sensor. (**b**) Magnified view of a single wrist pulse waveform. (**c**) The Fast Fourier transform of waves of the wrist pulse.

Besides the monitoring of human activities, heartbeat rate is also an important parameter related to human health. Due to the high sensitivity of the as-prepared sensor, the calculation of a human’s heartbeat rate can be achieved by detecting the human wrist pulse. To detect the pulse signal effectively and accurately, the fabricated sensor was tightly fixed to the radial artery on the wrist of the tester with commercial tape. The relative resistance change of the fabricated sensor exhibited a fast and clear response to the wrist pulse, as displayed in Figure 7a. One of the pulse waveforms is enlarged and displayed in Figure 7b. Three characteristic peaks of the pulse waveform can be clearly identified, which can be assigned to the percussion wave (P-wave), tidal wave (T-wave), and diastolic wave (D-wave) [31,32,33]. According to Figure 7a, the average time of one heartbeat is about 0.86 s. Based on this result, it can be calculated that the tester’s heart rate is about 70 beats per minute. In addition, the frequency spectrum of the corresponding pulse waveform is also presented in Figure 7c via a fast Fourier transform analysis. It is obvious that the main frequency component of the waveform is 1.16 Hz, which is consistent with the result of the above heartbeat calculation. Due to the accurate measurement of the pulse waveform by the sensor, the human heart rate can be calculated, combined with the above-mentioned monitoring of human limb movement, thereby realizing human health monitoring.

## 4. Conclusions

Unlike conventional flat electrospun films, three-dimensional microstructures were introduced to electrospun PU films directly during the electrospinning process and the films were coated with a silver conductive layer, thus obtaining conductive PU/Ag films with a rough surface. By placing two polyurethane/silver films face-to-face and encapsulating them with PDMS, a flexible piezoresistive pressure sensor based on microstructured PU nanofibers film was assembled. The working mechanism of this pressure sensor is the change in contact resistance between two PU/Ag films in response to the loading pressure. The assembled sensor exhibits high sensitivity, fast response time, and long-term stability due to the flexibility of the electrospun polyurethane films and the abundant conductive pathways generated by their unique surface microstructure. It was also proved that the as-prepared piezoresistive sensor could be applied to the detection of human joint movements and wrist pulses. The efficient approach we present here to fabricate the rough electrospun nanofiber film and flexible piezoresistive pressure sensor may be valuable for future wearable devices which are applicable in human health monitoring.

## Figures and Tables

**Table 1:** A Comparison of some piezoresistive pressure sensors reported recently with the proposed sensor.

Active Material	Sensing Mechanism	Maximum Sensitivity	Stability	Ref.
PVA/H_2_SO_4_@PU composite	Piezoresistive	8.0 kPa^−1^	1325 cycles	[17]
F-rGO@CNTs/CS aerogel	Piezoresistive	4.97 kPa^−1^	1000 cycles	[18]
Microstructured PDMS films	Piezoresistive	0.54 kPa^−1^	N/A	[28]
TPU/Carbon Black	Piezoresistive	5.54 kPa^−1^	10,000 cycles	[29]
carbonized melamine sponge/silicone rubber	Piezoresistive	0.635 kPa^−1^	~2000 cycles	[30]
Microstructured PU/Ag film	Piezoresistive	10.53 kPa^−1^	10,000 cycles	This work

## Data Availability

Data sharing is not applicable to this article.

## Supplementary Materials

The following supporting information can be downloaded at: www.mdpi.com/article/10.3390/nano12040723/s1, Figure S1: The photograph of the electrospinning unit; Figure S2: Photographs of (a) PDMS, (b) PU/Ag film, and (c) the fabricated piezoresistive pressure sensor at a bending state. Figure S3: An SEM image of the electrospun PU nanofibers film. The inset is a photograph of the film fixed on the sample holder with carbon tape. Figure S4: A photograph of the electrospun PU nanofibers film. Figure S5: A high magnification cross-section SEM image of PU/Ag film. Table S1: A comparison of other types of sensors reported recently with the proposed sensor. References [34,35,36,37,38,39,40,41,42,43] are cited in the supplementary materials.
