# Supplementary material for: Flexible Piezoresistive Pressure Sensor Based on Electrospun Rough Polyurethane Nanofibers Film for Human Motion Monitoring
Source: Nanomaterials (Basel). 2022 Feb 21;12(4):723. doi: 10.3390/nano12040723 (PMC8879922; doi:10.3390/nano12040723)
Supplement: Supplementary file 1 [file nanomaterials-12-00723-s001.zip › nanomaterials-1560560-supplementary-final.pdf]

nanomaterials

Supplementary materials

Flexible Piezoresistive Pressure Sensor Based on Electrospun Rough Polyurethane Nanofibers Film for Human Motion Monitoring

Bin Xue, Haiyi Xie, Jinxu Zhao, Jianming Zheng * and Chunye Xu *

Hefei National Laboratory for Physical Sciences at the Microscale, Department of Polymer Science and Engineering, University of Science and Technology of China, Hefei 230026, China; binxue@mail.ustc.edu.cn (B.X.); xiehy@mail.ustc.edu.cn (H.X.); zhaojinxu@mail.ustc.edu.cn (J.Z.)
* Correspondence: jmz@ustc.edu.cn (J.Z.); chunye@ustc.edu.cn (C.X.)

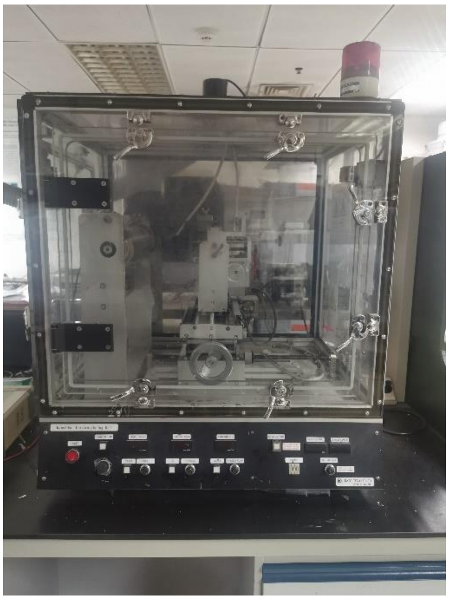

Figure S1. The photograph of the electrospinning unit.

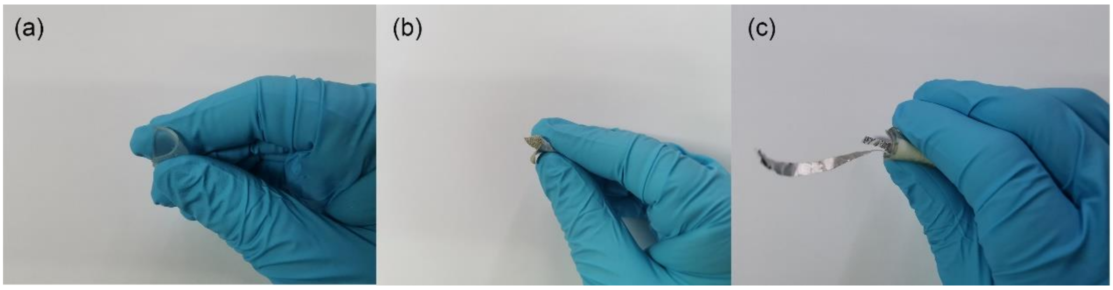

Figure S2. Photographs of (**a**) PDMS, (**b**) PU/Ag film, and (**c**) the fabricated piezoresistive pressure sensor at a bending state.

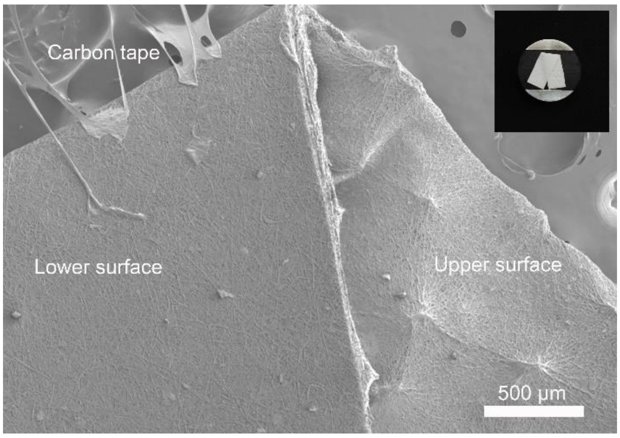

Figure S3. An SEM image of the electrospun PU nanofibers film. The inset is a photograph of the film fixed on the sample holder with carbon tape.

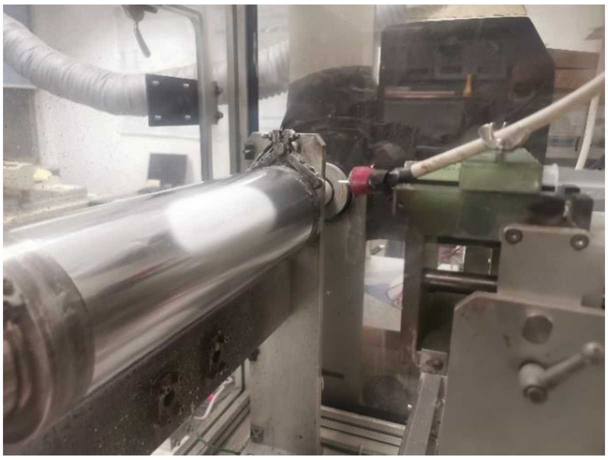

Figure S4. A photograph of the electrospun PU nanofibers film.

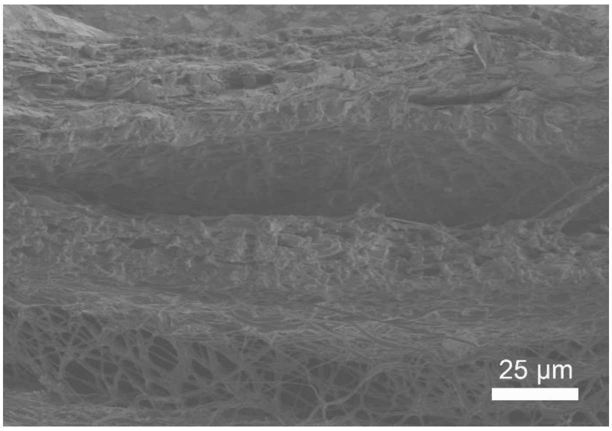

Figure S5. A high magnification cross-section SEM image of PU/Ag film.

Table S1. A comparison of other types of sensors reported recently with the proposed sensor.

Active material	Sensing mechanism	Maximum sensitivity	Stability	Ref.
PI-MWCNTs/microstructured PDMS	Capacitance	1.3 kPa^{-1}	> 4000 cycles	[34]
PDMS/PVDF $BaTiO_3$ filler	Capacitance	4.9 kPa^{-1}	5000 cycles	[35]
PDMS	Capacitance	44.5 kPa^{-1}	5000 cycles	[36]
ITO coated PET/porous PDMS	Capacitance	0.095 kPa^{-1}	N/A	[37]
porous PDMS/CNT film	Capacitance	2.51 kPa^{-1}	5000 cycles	[38]
BCZT/PDMS	Piezoelectric	~0.55 V kPa^{-1}	2×10^6 cycles	[39]
Hierarchical ZnO NRs/PVDF	Piezoelectric	11.07 mV kPa^{-1}	3600 cycles	[40]
ZnO@C/PVDF	Piezoelectric	0.98 V kPa^{-1}	4000 cycles	[41]
MXene/PVDF hybrid film	Piezoelectric	0.0480 V N^{-1}	N/A	[42]
PDA@BTO/PVDF	Piezoelectric	0.38 V N^{-1}	6834 cycles	[43]
Microstructured PU/Ag film	Piezoresistive	10.53 kPa^{-1}	10000 cycles	This work

References

34. Chen, Y.; Zhang, P.; Li, Y.; Zhang, K.; Su, J.; Huang, L. Flexible capacitive pressure sensor based on multi-walled carbon nanotubes microstructure electrodes. *J. Phys. D Appl. Phys.* **2021**, *54*, 155101.
35. Ma, L.; Yu, X.; Yang, Y.; Hu, Y.; Zhang, X.; Li, H.; Ouyang, X.; Zhu, P.; Sun, R.; Wong, C.-p. Highly sensitive flexible capacitive pressure sensor with a broad linear response range and finite element analysis of micro-array electrode. *J. Mater.* **2020**, *6* , 321–329.
36. Yang, J.C.; Kim, J.O.; Oh, J.; Kwon, S.Y.; Sim, J.Y.; Kim, D.W.; Choi, H.B.; Park, S. Microstructured Porous Pyramid-Based Ultrahigh Sensitive Pressure Sensor Insensitive to Strain and Temperature. ACS Appl. Mater. Interfaces **2019**, *11*, 19472–19480.
37. Bijender; Kumar, A. Flexible and wearable capacitive pressure sensor for blood pressure monitoring. *Sens. Bio-Sens. Res.* **2021**, *33*, 100434.
38. Zhang, Z.; Gui, X.; Hu, Q.; Yang, L.; Yang, R.; Huang, B.; Yang, B.R.; Tang, Z. Highly Sensitive Capacitive Pressure Sensor Based on a Micropyramid Array for Health and Motion Monitoring. *Adv. Electron. Mater.* **2021**, *7*, 2100174.
39. Gao, X.; Zheng, M.; Yan, X.; Fu, J.; Zhu, M.; Hou, Y. The alignment of BCZT particles in PDMS boosts the sensitivity and cycling reliability of a flexible piezoelectric touch sensor. *J. Mater. Chem. C* **2019**, *7*, 961–967.
40. Feng, W.; Chen, Y.; Wang, W.; Yu, D. A waterproof and breathable textile pressure sensor with high sensitivity based on PVDF/ZnO hierarchical structure. *Colloids Surf. A* **2022**, *633*, 127890.
41. Li, X.; Ji, D.; Yu, B.; Ghosh, R.; He, J.; Qin, X.; Ramakrishna, S. Boosting piezoelectric and triboelectric effects of PVDF nanofiber through carbon-coated piezoelectric nanoparticles for highly sensitive wearable sensors. *Chem. Eng. J.* **2021**, *426*, 130345.
42. Zhao, Q.; Yang, L.; Ma, Y.; Huang, H.; He, H.; Ji, H.; Wang, Z.; Qiu, J. Highly sensitive, reliable and flexible pressure sensor based on piezoelectric PVDF hybrid film using MXene nanosheet reinforcement. *J. Alloys Compd.* **2021**, *886*, 161069.
43. Su, Y.; Li, W.; Yuan, L.; Chen, C.; Pan, H.; Xie, G.; Conta, G.; Ferrier, S.; Zhao, X.; Chen, G.; et al. Piezoelectric fiber composites with polydopamine interfacial layer for self-powered wearable biomonitoring. *Nano Energy* **2021**, *89*, 106321.
